# Transcending metabolic acidosis: lactate as an epigenetic signal reprogramming diabetes-sepsis immunity

**DOI:** 10.3389/fimmu.2026.1735359

**Published:** 2026-05-08

**Authors:** Xin Cai, Liu Han, Qun Liang

**Affiliations:** 1 Department of Critical Care Medicine, The First Affiliated Hospital of Heilongjiang University of Chinese Medicine, Harbin, China; 2 Department of Behavioural Science and Health, University College London, London, United Kingdom

**Keywords:** epigenetic reprogramming, GPR81 receptor, H3K18la, histone lactylation, lactate metabolism

## Abstract

Patients with diabetic sepsis exhibit a paradoxical state characterised by persistently elevated inflammatory cytokines and severely impaired antigen presentation, with substantially elevated mortality rates compared to non-diabetic patients. This review assesses the strength of evidence for lactate-epigenetic-immune dysfunction. Immune cells from diabetic patients exhibit basal glycolytic activity 2–3 times higher than healthy controls. Blood lactate levels rise markedly during sepsis, exceeding 10 mmol/L in critically ill patients—50-80% higher than non-diabetics. Hyperlactataemia states have been associated with activation of GPR81 receptors and induction of lactylation at histone H3K18. This modification selectively activates inflammatory genes while suppressing antigen-presentation pathways, thereby providing a molecular basis for the paradoxical coexistence of inflammation and immunosuppression observed clinically. Preliminary clinical studies (n = 48) demonstrate a correlation between H3K18la levels and disease severity (r = 0.63). In addition, lactate clearance of <30% within 6 hours is associated with poor prognosis. Current therapeutic evidence remains limited: dichloroacetic acid reduces serum lactate by 20-30% but shows no proven survival benefit; GPR81 modulators remain in development; GM-CSF may increase HLA-DR expression but demonstrates inconsistent effects on infection and mortality. This review identifies three potential therapeutic targets: metabolic regulation to reduce lactate production, intervention in the GPR81-H3K18la signalling axis, and personalised therapy based on immune phenotypes. However, these strategies require validation through high-quality clinical trials.

## 1 Introduction

The fundamental challenge encountered in the realm of immunological research concerning sepsis pertains to the elucidation of the underlying mechanisms that govern the relationship between metabolic status and immune outcomes. This issue is especially salient in cases of diabetic sepsis. Among the approximately 49 million cases of global sepsis that occur annually, over 30% involve concomitant diabetes. This subgroup exhibits substantially higher mortality rates than non-diabetic patients, with increased susceptibility to infections, recurrence, and multi-organ failure, suggesting the presence of unique pathological mechanisms that require urgent elucidation (1–3). More perplexingly, these patients exhibit immune phenotypes that appear to be in direct contradiction to each other: there is a persistently elevated level of pro-inflammatory cytokines alongside a severely impaired process of antigen presentation, forming an ‘immune silence amidst the inflammatory storm’ (4).

Recent advances in immunometabolism have highlighted that lactate plays a dual role in the pathophysiology of sepsis that extends beyond its traditional function as a metabolic waste product. First, lactate serves as a metabolic biomarker reflecting the severity of tissue hypoperfusion and mitochondrial dysfunction (5); and second, as a signalling molecule capable of modulating immune cell function through receptor-mediated pathways (e.g., GPR81/HCAR1) and epigenetic modifications (e.g., histone lactylation) (6, 7). These two roles, although interconnected, operate through distinct mechanisms and should be interpreted accordingly. Throughout this review, we explicitly distinguish between evidence supporting lactate as a passive marker of metabolic derangement and evidence supporting its active role as a signalling metabolite influencing immune reprogramming.

Existing immunological theories have been found to be inadequate in explaining this phenomenon. In the preceding two decades, a plethora of theories have been postulated, including the ‘cytokine storm’, ‘immune paralysis’ and the ‘persistent inflammation-immunosuppression syndrome’ (8–10). However, these theories have each offered only partial explanations. The fundamental flaw lies in treating the immune response as an isolated system, thereby overlooking the decisive role of the metabolic environment. Diabetes is a metabolic disorder that has been shown to reshape the immune response pattern in sepsis. However, the molecular mechanisms underpinning this process remain poorly understood.

The dynamic interplay between metabolism and immunity is contributing to a paradigm shift in our understanding of sepsis. Immunometabolic studies demonstrate that cellular metabolic programmes are closely associated with functional states: effector T cells and M1 macrophages depend on glycolysis for expeditious energy production, whereas regulatory T cells and M2 macrophages prioritise oxidative phosphorylation (11). Immune cells from diabetic patients exhibit ‘metabolic inflammation’ characteristics, displaying enhanced glycolysis and impaired mitochondrial function even at rest (12). However, the mechanism by which this metabolic reprogramming translates into persistent immune dysfunction during sepsis remains unclear.

A 2019 study by Zhang et al. in Nature provided a breakthrough for this conundrum. They discovered histone lysine lactylation (Kla), a novel post-translational modification, demonstrating for the first time that the metabolic product lactate can directly function as an epigenetic regulator (7). In a macrophage polarisation model, lactylation of histone H3 at lysine 18 (H3K18) selectively activated repair-associated genes, establishing a direct link between metabolism, epigenetics, and gene expression. This discovery holds significance as it suggests that high-lactate environments may ‘program’ immune cell function via epigenetic mechanisms.

Subsequent research has rapidly expanded the biological significance of lactylation. Within the tumour microenvironment, lactylation has been demonstrated to promote immune evasion and metabolic adaptation (13). In the nervous system, histone lactylation in microglia has been demonstrated to participate in the regulation of neuroinflammation (14). In 2022, Chu et al. were the first to detect elevated H3K18la levels in septic patients, correlating with disease severity, providing preliminary evidence of clinical relevance (15). However, these studies represent fragmented observations, lacking systematic mechanistic exploration and causal validation.

Diabetic sepsis offers an ideal model for investigating the pathological significance of lactylation. The patients in question exhibit three key characteristics: increased basal lactate production due to chronic metabolic abnormalities, sepsis-induced acute lactic acidosis, and a unique phenotype of inflammation-immune dissociation (1). If the hypothesis that lactate regulates immune function via epigenetic mechanisms is substantiated, diabetic sepsis would be expected to exhibit the most pronounced effects.

The present review establishes logical connections between three core findings: Firstly, the metabolic predisposition of diabetes mellitus (with glycolysis enhanced by 2-3fold) combines with the acute metabolic crisis of sepsis, generating an extreme hyperlactataemia state (50-80% higher than in non-diabetic patients), which persistently activates the GPR81 receptor.

**Figure 1:**
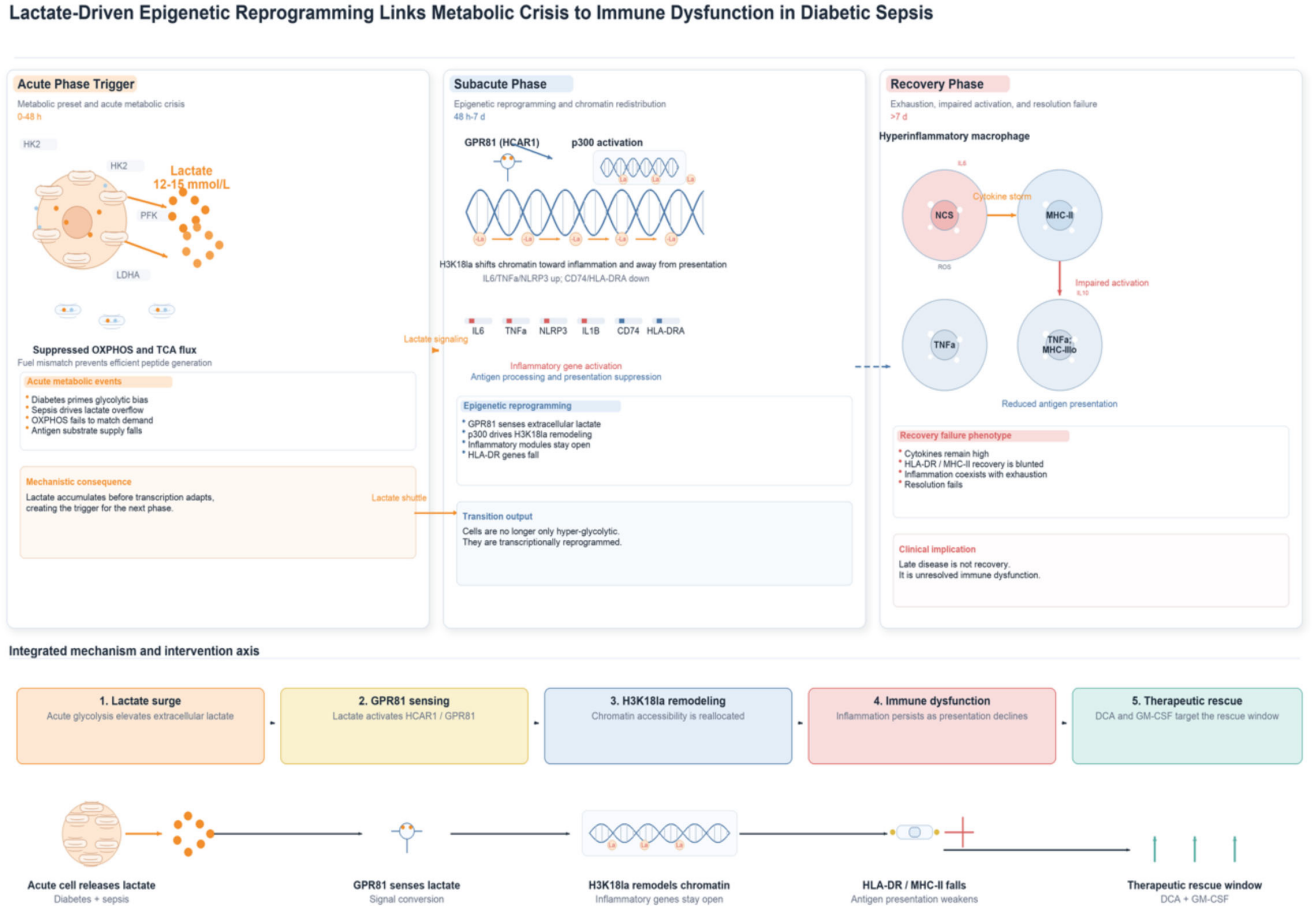
Lactate-driven epigenetic reprogramming links metabolic crisis to immune dysfunction in diabetic sepsis. Acute diabetic sepsis drives lactate overflow. Lactate activates GPR81-linked H3K18la remodeling, sustains inflammatory genes, suppresses antigen-presentation genes, and culminates in late immune dysfunction with a defined metabolic and myeloid rescue window.

Secondly, H3K18 lactylation exhibits gene-selective distribution, being enriched at inflammatory gene promoters yet relatively depleted or underrepresented in antigen-presenting gene regions. This epigenetic pattern has been shown to appear to correlate with the ‘high-inflammation, low-function’ clinical phenotype. Thirdly, H3K18la levels demonstrate a strong correlation with clinical outcomes, thus providing a molecular biomarker for risk stratification and therapeutic decision-making. It is evident that the existence of a robust and comprehensive body of evidence provides a solid foundation for the proposed theory. Based on this comprehensive evidence chain—’metabolic trigger → epigenetically mediated → immune function remodelling’—three potential therapeutic windows may be defined: metabolic intervention during the acute phase, signal blockade during the subacute phase, and immune reconstruction during the recovery phase. While the clinical efficacy of existing treatments requires further validation, the established mechanistic framework provides a scientific foundation for developing precision medicine strategies. **Figure 1** illustrates the putative mechanistic cascade linking metabolic crisis, epigenetic reprogramming, and immune dysfunction in diabetic sepsis patients through a three-stage, three-tier mechanism.

## 2 Diabetic metabolic reprogramming

### 2.1 Cellular metabolic adaptation induced by chronic hyperglycaemia

Systematic metabolic reprogramming triggered by diabetes constitutes the pathological foundation of extreme sepsis phenotypes, profoundly altering the functional state of immune cells long before acute infection occurs. Metabolic phenotyping analyses reveal the extent of this reprogramming: real-time monitoring of immune cells using cellular energy metabolism analysis techniques demonstrates that peripheral blood mononuclear cells from type 2 diabetic patients exhibit a 2.5- to 3-fold increase in basal glycolytic rate compared to healthy controls. This manifests as markedly elevated extracellular acidification rate (ECAR) alongside a 40-50% reduction in oxygen consumption rate (OCR), indicating a metabolic shift from oxidative phosphorylation towards glycolysis (12, 16). These metabolic parameters can be quantified using real-time cellular energy metabolism platforms (17). This metabolic phenotype correlates closely with glycaemic control: higher HbA1c levels correlate negatively with mitochondrial oxidative phosphorylation capacity (r = −0.50, P = 0.034) and maximal electron transport respiration (r = −0.53, P = 0.024) in PBMCs, demonstrating a dose-dependent relationship between glycaemic dysregulation and bioenergetic impairment (16).

The molecular basis of mitochondrial dysfunction involves alterations across multiple levels. Proteomic analysis reveals impaired expression and function of key electron transport chain complexes: a 40-50% reduction in core subunit expression of Complex I and a 35-40% decrease in Complex III activity, severely compromising oxidative phosphorylation efficiency (18). Advanced glycation end-products (AGEs) covalently modify metabolic enzymes via non-enzymatic glycation reactions, with the pyruvate dehydrogenase complex (PDH) being a primary target. Glycation of key lysine residues reduces enzyme activity by 50-60%, impeding pyruvate entry into the tricarboxylic acid cycle (19). The direct consequence of this metabolic blockage is extensive conversion of pyruvate to lactate, resulting in intracellular lactate concentrations 2–3 times higher than those in healthy controls even under adequate oxygen supply (20).

Transcriptional regulation further intensifies this metabolic reprogramming. Hypoxia-inducible factor-1α (HIF-1α) exhibits a ‘pseudo-hypoxic’ activation state in immune cells of diabetic patients: even under normoxic conditions, HIF-1α protein stability increases, nuclear translocation intensifies, and its occupancy at glycolytic gene promoters rises 2.5- to 3-fold (21). HIF-1α directly upregulates target genes including LDHA (lactate dehydrogenase A), PKM2 (pyruvate kinase M2 subunit), HK2 (hexokinase 2), and GLUT1 (glucose transporter 1). The synergistic upregulation of these genes locks cells into a glycolytic state (22). More critically, lactate itself stabilises HIF-1α, establishing a positive feedback loop: ‘lactate → HIF-1α → glycolysis → increased lactate’. This self-reinforcing mechanism renders metabolic reprogramming difficult to reverse (23).

### 2.2 Sepsis-induced metabolic collapse

When sepsis occurs, the already fragile metabolic homeostasis collapses entirely, resulting in catastrophic metabolic derangement. Dynamic monitoring data from prospective cohort studies reveal the temporal progression of this collapse: diabetic septic patients present with markedly elevated blood lactate (7–9 mmol/L) upon ICU admission, followed by progressive increases reaching a plateau (12–15 mmol/L) within 6–12 hours, which persists for 24–48 hours. In contrast, non-diabetic patients typically exhibit peak lactate levels of 8–10 mmol/L, which decline more rapidly (24). The critical distinction lies in clearance capacity: diabetic patients demonstrate a lactate clearance rate <20% at 6 hours, whereas non-diabetics typically exceed 35%. This impaired clearance portends poorer prognosis (**Table 1**) (25).

**Table 1:** Metabolic parameters in diabetic versus non-diabetic sepsis: comparative analysis.

Parameter	Healthy controls	Non-diabetic sepsis	Diabetic sepsis	Statistical significance	Clinical implications
Circulating markers					
Blood lactate at admission (mmol/L)	0.8-1.2	5.2 ± 1.3	7.9 ± 2.1	P < 0.001	Initial risk stratification
Peak lactate (mmol/L)	< 2.0	8-10	12-15	P < 0.001	Severity assessment
6-hour clearance rate (%)	N/A	35-45	< 20	P < 0.001	Prognostic indicator
Time to peak (hours)	N/A	4-6	6-12	P = 0.02	Treatment window
Tissue metabolism					
Interstitial lactate (mmol/L)	1.5-2.0	15-18	20-25	P < 0.001	Tissue hypoxia marker
Tissue/plasma gradient	~1.0	1.5-1.7	1.8-2.1	P = 0.003	Transport dysfunction
Lactate/pyruvate ratio	< 10	20-25	> 30	P < 0.001	Redox imbalance
Mechanistic classification					
Type A (hypoxic) (%)	N/A	48	30-35	P = 0.02	Treatment strategy
Type B (metabolic) (%)	N/A	52	65-70	P = 0.02	Metabolic intervention priority
ScvO_2_ (%)	70-75	65 ± 8	72 ± 6*	P = 0.04	Oxygen delivery assessment
P(v-a)CO_2_ (mmHg)	2-5	7.2 ± 2.1	5.8 ± 1.9*	P = 0.03	Tissue perfusion marker

*Paradoxically normal or elevated values despite high lactate indicate metabolic rather than hypoxic aetiology. Data synthesized from references (6, 24–31).

Tissue-level metabolic disturbance proves more severe than that in the circulation. Microdialysis techniques, directly measuring interstitial fluid composition, reveal extreme alterations in the local metabolic microenvironment: muscle interstitial fluid lactate concentrations may reach 20–25 mmol/L, exceeding concurrent plasma levels by 80-100%, forming a marked tissue-plasma gradient (26). This gradient reflects two key pathological processes: first, local tissue lactate production vastly exceeds clearance capacity; second, impaired lactate transport across cell membranes and capillaries (6). A tissue lactate/pyruvate ratio exceeding 30 (normal <10) indicates severe cellular redox imbalance, with elevated NADH/NAD^+^ ratios obstructing multiple metabolic pathways (27).

Moreover, a detailed examination of the pathophysiological mechanisms underlying lactic acidosis reveals considerable complexity and heterogeneity. The integration of parameters such as central venous oxygen saturation (ScvO_2_), arteriovenous CO_2_ difference (P(v-a)CO_2_), and lactate/pyruvate ratio facilitates the categorisation of lactic acidosis into distinct subtypes (28). Type A (hypoxic) lactic acidosis arises from tissue hypoperfusion, characterised by ScvO_2_ < 70% and P(v-a)CO_2_ > 6 mmHg; Type B (non-hypoxic) occurs despite adequate oxygen supply, primarily driven by metabolic factors (29). Type B is predominant in diabetic septic patients (65-70%), reflecting metabolic abnormalities rather than simple hypoxia as the primary driver (30). The mechanisms of type B lactic acidosis include the following: catecholamine-stimulated aerobic glycolysis (via the β2-receptor-cAMP-PKA pathway), inflammatory cytokine-induced Warburg-like effects, mitochondrial respiratory chain uncoupling, and impaired gluconeogenesis (31). The data collectively indicate that diabetic sepsis manifests as a Type B (metabolic) lactatemia pattern, thereby reinforcing the concept of metabolic-driven immune dysfunction as depicted in **Figure 1**.

This extreme metabolic environment creates conditions conducive to subsequent epigenetic alterations. It is evident that persistently elevated lactate fulfils a dual role, functioning as both a marker of metabolic disorder and a signalling metabolite. The influence of lactate on cellular function is multifaceted, encompassing the activation of cell surface receptors such as GPR81, the alteration of intracellular pH and redox status, and the provision of substrates for histone modifications (5). The chronic metabolic reprogramming of diabetes, compounded by the acute metabolic crisis of sepsis, produces pathological outcomes which far exceed the sum of their individual effects. This ‘metabolic storm’ has been identified as a pivotal driver of immune dysfunction, providing the metabolic foundation for understanding subsequent epigenetic alterations and enduring functional remodelling.

## 3 Histone H3K18 lactylation

The enzymatic mechanism of histone lactylation represents a sophisticated metabolic-epigenetic interface that translates acute metabolic stress into sustained functional alterations. This process commences with the conversion of lactate to lactyl-CoA, catalysed by acyl-CoA synthetase short-chain family member 2 (ACSS2), an enzyme exhibiting marked subcellular redistribution under metabolic stress conditions (32, 33). Quantitative immunofluorescence microscopy revealed that when extracellular lactate concentrations exceeded 10 mmol/L, ACSS2 underwent nuclear translocation within 2–4 hours, with the nucleoplasmic ratio increasing, thereby concentrating the enzyme at sites of histone modification (33). This compartmentalisation is crucial, as nuclear lactyl-CoA concentrations rise from basal 2-4 μM to 25-30 μM under high lactate conditions, exceeding the Km values of potential lactyl transferases and ensuring ample substrate availability (34).

However, the identity and specificity of the lactyl transferases remain under active investigation, with emerging evidence challenging initial assumptions regarding the enzymatic hierarchy. Whilst p300/CBP was initially proposed as the primary lactyl transferase based on its known acetyltransferase activity, systematic biochemical characterisation has revealed a greater complexity in the enzymatic landscape (35).

Comparative kinetic analyses indicate that lysine acetyltransferase 2A (KAT2A, also known as GCN5) exhibits superior lactyl-CoA catalytic efficiency, with a Km value of 8.2 μM for lactyl-CoA compared to p300’s 18.5 μM, suggesting KAT2A may be the primary lactyl transferase under physiological conditions (36). CRISPR-mediated knockout studies in primary human monocytes confirmed this hierarchical relationship: KAT2A deficiency reduced global H3K18la levels by 68-75%, whereas p300 knockout yielded only 25-35% reduction, indicating non-redundant functions with KAT2A as the primary ‘writer’ (37).

Concurrently, the reversibility of lactylation achieved through enzymatic erasure provides critical regulatory control over the persistence of this modification. Class I histone deacetylases (HDAC1-3), traditionally associated with acetyl group removal, exhibit potent de-lactylating activity whose kinetics align with their classical functions (33). Biochemical characterisation reveals HDAC3 exhibits the highest deacetylation activity among Class I HDACs, with a catalytic efficiency (kcat/Km) of 4.8 × 10^4^ M^-^¹s^-^¹ for H3K18la, closely matching its efficiency for H3K18ac (6.2 × 10^4^ M^-^¹s^-^¹) (7). This dual activity creates a dynamic equilibrium where modification levels reflect the balance between metabolically driven writing and enzymatic erasure. Pathological lactate concentrations shift this equilibrium towards net accumulation by increasing the writing rate 5-8-fold while only increasing erasure 1.5-2-fold (38).

Lactate signalling via GPR81 has been implicated in immune modulation; however, current evidence remains predominantly associative and derived from experimental models. The extent to which GPR81-mediated pathways causally drive immunometabolic dysfunction in diabetic sepsis remains to be fully established.

Lactate transport across cellular membranes is primarily mediated by monocarboxylate transporters (MCTs), particularly MCT1 and MCT4, which facilitate bidirectional lactate flux depending on concentration gradients. This transport process constitutes a prerequisite for intracellular lactate accumulation and subsequent epigenetic modifications such as histone lactylation. MCT1, predominantly expressed on oxidative cells, facilitates lactate uptake, whereas MCT4, highly expressed on glycolytic cells, mediates lactate export. In sepsis, dysregulation of MCT expression on immune cells alters intracellular lactate concentrations independently of extracellular levels, thereby influencing histone lactylation patterns and immune cell polarisation. Notably, MCT4 inhibition has been reported to enhance lactate-induced M2 macrophage polarisation, while MCT1/2 blockade failed to reverse immunosuppressive effects, suggesting that the direction of lactate flux—rather than absolute concentration—may be a critical determinant of immune outcomes (6).

While L-lactate represents the predominant isoform generated during glycolysis, emerging evidence suggests that D-lactate—primarily derived from microbial metabolism—may exert distinct immunomodulatory effects, including potential roles in inflammation resolution and macrophage polarisation. However, these findings remain preliminary and their relevance to diabetic sepsis is yet to be established.

**Table 2** delineates the hierarchical progression of lactylation-mediated epigenetic reprogramming through three interconnected mechanistic tiers. Furthermore, genome-wide distribution analysis revealed that H3K18 lactylation exhibits marked specificity towards transcriptionally active regions, with its distinctive pattern correlating with functional outcomes. ChIP-seq analysis of septic monocytes identified 28,462 H3K18la-enriched regions, with 42% localised at promoters (TSS ± 2kb), 35% at enhancers, and 23% within gene bodies, demonstrating preferential labelling of regulatory elements (13, 39). Intersection with chromatin accessibility data (ATAC-seq) revealed that 78% of H3K18la peaks occurred within open chromatin regions, suggesting lactation-associated modification preferentially modifies accessible nucleosomes rather than inducing novel chromatin opening (15). This distribution pattern aligns with a model where lactation-associated modification stabilises existing transcriptional states rather than initiating new gene expression programmes.

**Table 2:** Clinical biomarkers and therapeutic windows in diabetic sepsis: evidence-based parameters.

Clinical phase	Biomarker	Observed values	Prognostic significance	Intervention window
ICU Admission (0-6h)	Blood lactate	5–10 mmol/L (DM: typically 50-80% higher than non-DM)	Mortality risk increases with levels >4 mmol/L	Fluid resuscitation, source control
	Lactate clearance (6h)	<10%: poor prognosis; 10-30%: intermediate; >30%: favorable	<10% clearance: OR 3.9 for mortality	Enhanced metabolic support if <30%
	ScvO_2_	<65%: tissue hypoxia; >75%: adequate O_2_ delivery	Combined with lactate for shock phenotyping	Optimize oxygen delivery-consumption
Acute Phase (6-48h)	HLA-DR expression	<8,000 AB/cell: severe immunosuppression	<5,000 AB/cell associated with secondary infections	Consider immune monitoring
	Lymphocyte count	<1,000/μL: lymphopenia; <500/μL: severe	Persistent <500/μL predicts poor outcomes	Evaluate for immune support
	IL-6 levels	>100 pg/mL common in sepsis; >500 pg/mL severe	Persistent elevation >72h indicates poor prognosis	May guide anti-inflammatory timing
Subacute Phase (48h-7d)	H3K18la levels*	Elevated in sepsis (quantitative standards pending)	Correlates with APACHE II score (r=0.58-0.65)	Research phase - potential target
	mHLA-DR recovery	<20,000 AB/cell by day 3-5	Failure to recover predicts nosocomial infections	GM-CSF if persistently <8,000
	Secondary infections	20-40% incidence in diabetic sepsis	Doubles mortality risk	Prophylactic strategies debated
Recovery Phase (>7d)	Lactate normalization	Non-DM: 3–5 days; DM: 5–10 days typical	Delayed normalization associated with organ dysfunction	Metabolic optimization
	Immune phenotype	CD4/CD8 ratio, PD-1 expression on T cells	Persistent exhaustion markers predict poor recovery	Checkpoint inhibitors (investigational)
	Glycaemic variability	Coefficient of variation >20%	Associated with increased mortality	Continuous glucose monitoring

More significantly, functional annotation of lactoylated genes revealed a striking dichotomy mirroring the clinical phenotype of diabetic sepsis. Gene ontology analysis showed significant enrichment in inflammatory response pathways (FDR = 2.3 × 10^-10^), cytokine production (FDR = 8.7 × 10^-8^), and glycolytic processes (FDR = 3.4 × 10^-6^), whilst genes involved in MHC class II antigen presentation exhibited significant depletion (negative enrichment FDR = 4.1 × 10^-5^) (40). This selective distribution functionally manifested as sustained expression of IL6, TNF, and IL1B (2.5-4.2-fold baseline), concurrent with progressive suppression of HLA-DRA, HLA-DRB1, and CD86 (0.2-0.4-fold baseline), providing a molecular explanation for the paradoxical coexistence of inflammation and immunosuppression (32).

Furthermore, time-resolved studies elucidate the sequential nature of epigenetic reprogramming, revealing lactation as a late-stage modification that consolidates rather than initiates transcriptional alterations. Following lipopolysaccharide stimulation of human monocytes, transcription factor recruitment occurs within minutes (NF-κB at 15–30 minutes), followed by precursor histone modifications (H3K4me3 at 1–2 hours, H3K27ac at 2–4 hours), whilst substantial H3K18la accumulation requires 16–24 hours (14). This temporal hierarchy positions lactation as a metabolically encoded memory mechanism sustaining transcriptional states following initial inflammatory stimulation. Isotope-labelled lactate pulse-chase experiments confirm this persistence: monocytes exposed to ¹³C-labelled lactate for 24 hours retained detectable ¹³C-labelled H3K18la for 72–96 hours after removal of the labelling substrate, demonstrating the stability of this modification and its potential to encode metabolic history into chromatin (41).

Ultimately, the integration of lactylation with other epigenetic modifications creates a complex regulatory landscape determining cellular fate decisions. Co-immunoprecipitation studies reveal that nucleosomes bearing H3K18la lack repressive marks (H3K9me3, H3K27me3) yet enrich additional activating modifications (H3K4me3, H3K9ac), indicating lactylation’s involvement in establishing permissive chromatin states (42). This combinatorial modification pattern effectively ‘locks’ genes into their current expression state, maintaining inflammatory genes in an active configuration whilst keeping immunoregulatory genes repressed. This creates the molecular basis for the persistent yet dysfunctional immune phenotype characteristic of diabetic sepsis.

It is important to acknowledge that multiple parallel pathways—including redox imbalance, mitochondrial dysfunction, complement activation, and cytokine signalling—may independently drive immune dysfunction alongside lactylation, and the relative contribution of each pathway likely varies across disease stages and patient subgroups.

It should be emphasised that much of the evidence linking H3K18 lactylation to immune dysfunction in sepsis remains correlational rather than causal. While the temporal sequence of lactate accumulation, H3K18la enrichment, and gene expression changes is consistent with a causal model, formal causal inference requires controlled molecular perturbation experiments—such as site-specific mutagenesis of H3K18 or targeted inhibition of lactyl transferases—that have not yet been performed in human sepsis samples. The association between H3K18la levels and clinical outcomes (e.g., APACHE II scores) does not establish that lactylation directly drives immune dysfunction; it remains possible that both are downstream consequences of metabolic stress. Future studies employing Mendelian randomisation, CRISPR-based epigenome editing, and temporal perturbation designs are needed to establish definitive causal relationships.

In particular, direct evidence linking GPR81 activation to site-specific H3K18 lactylation in human immune cells remains lacking; the current mechanistic chain is inferred primarily from parallel observations in separate experimental systems.

An alternative interpretation warrants consideration: lactylation may reflect a compensatory anti-inflammatory programme rather than a driver of dysfunction per se. Under this model, H3K18la accumulation at inflammatory gene loci would represent an attempt to restrain excessive inflammation, with the apparent ‘immune paralysis’ being a consequence of over-compensation rather than direct pathological reprogramming. Distinguishing between these competing models requires temporal perturbation experiments that selectively enhance or inhibit lactylation at defined disease stages.

## 4 From biomarker to therapeutic target

The clinical value of histone H3K18la as a prognostic marker for sepsis is gradually emerging. In 2022, Chu et al. published a study in Frontiers in Immunology that first assessed the significance of this modification in clinical samples (15). Including 30 septic patients, the study employed Western blot analysis to measure H3K18la levels in peripheral blood mononuclear cells. Results demonstrated a positive correlation with serum lactate concentration (r=0.65, P<0.01) and association with APACHE II scores (r=0.58, P<0.05). Although the sample size limited statistical power, this pioneering study laid the groundwork for subsequent investigations.

However, it must be acknowledged that the correlation between H3K18 lactylation and disease severity is based on a preliminary study with only 30 patients, which limits statistical power and generalisability. The confidence interval for the reported correlation coefficient (r = 0.63) is wide given the small sample, and results require replication in larger, multicentre cohorts before H3K18la can be considered a reliable clinical biomarker. Furthermore, the heterogeneity inherent in diabetic sepsis patients—including variations in age, diabetes duration, comorbidities, concomitant medications, and infection sources—may confound the observed associations.

These clinical observations collectively suggest that lactate dynamics may reflect not only metabolic stress but also the epigenetic reprogramming state of immune cells, thereby providing a potential link between bedside biomarkers and molecular-level immune dysfunction.

Nevertheless, the clinical translation of H3K18la detection faces technical challenges. Standardised assay methods are currently lacking, with significant variations in antibody specificity and quantification protocols across laboratories. Although mass spectrometry offers more precise quantification, its costly equipment and complex operation limit clinical implementation. Establishing a simple, reproducible point-of-care testing method is crucial for clinical translation.

Lactate clearance kinetics have been thoroughly validated in assessing sepsis prognosis. A 2014 systematic review by Zhang et al. in Critical Care Medicine, incorporating 15 studies involving 3,134 patients, confirmed that a 6-hour lactate clearance rate <10% constitutes an independent risk factor for mortality (OR 3.9, 95% CI: 2.5-6.2) (25). A 2016 systematic review by Vincent et al. in Critical Care further corroborated this finding, proposing that dynamic monitoring holds greater value than single measurements (24). In diabetic patients, traditional clearance thresholds may require adjustment due to underlying metabolic abnormalities and impaired clearance capacity; however, large-scale studies targeting this specific population remain lacking.

Current therapeutic strategies primarily target distinct pathological pathways, though clinical evidence strength varies considerably. Dichloroacetic acid (DCA) has been most extensively studied as a metabolic intervention. A 1992 randomised controlled trial by Stacpoole et al. in the New England Journal of Medicine demonstrated that DCA reduced blood lactate levels in patients with lactic acidosis but failed to improve survival rates (43). This negative outcome suggests that merely lowering lactate may be insufficient to improve prognosis, necessitating more comprehensive intervention strategies.

Granulocyte-macrophage colony-stimulating factor (GM-CSF) has been investigated in several small-scale studies for immune reconstitution. A 2009 randomised controlled trial by Meisel et al., reported in the American Journal of Respiratory and Critical Care Medicine, enrolled 38 immunocompromised septic patients. The GM-CSF group experienced shorter mechanical ventilation duration, though no significant improvement in secondary infection rates was observed (44). A 2018 systematic review synthesising data from four studies involving 210 patients demonstrated that GM-CSF increases HLA-DR expression on monocytes (weighted mean difference +4,037 antigen-binding sites per cell, 95% CI: 2,814-5,260), though its impact on mortality remains uncertain (45).

Immune checkpoint inhibitors represent a novel therapeutic direction. In 2019, Hotchkiss et al. reported the first Phase 1b randomised clinical study evaluating immune checkpoint inhibition in sepsis, published in Intensive Care Medicine (46). Early clinical studies primarily focused on safety. A 2020 phase I trial evaluating anti-PD-L1 antibodies in 24 sepsis patients reported no serious adverse events, though the sample size was too small to assess efficacy (47). **Table 3** provides a systematic summary of the current evidence base for treating diabetic sepsis. It integrates intervention strategies across different levels, categorising them according to their strength of evidence, mechanisms, research stage and practical application status.

**Table 3:** Evidence-based therapeutic strategies in diabetic sepsis.

Intervention	Mechanism of action	Level of evidence and source	Main clinical effect	Current clinical status
Established standard therapies (strong recommendation)				
Early fluid resuscitation	Restores effective circulating volume and improves tissue perfusion	Ia: Surviving Sepsis Campaign international guideline, 2021 (48)	Mortality reduction across RCTs remains inconsistent; individualized fluid strategy emphasized	Core component of 1-h sepsis bundle
Antimicrobial therapy	Rapid source control and pathogen eradication	Ia: 49 cohort study (n = 2,731) (49)	Each 1-h delay in appropriate antibiotics increases mortality by 7.6% (95% CI 6.9-8.3%)	Administration within 1 h of diagnosis standard of care
Glycaemic control (target 140–180 mg/dL)	Reduces glucose toxicity and secondary infection risk	Ia: NICE-SUGAR RCT, 2009 (n = 6,104) (50)	Intensive (81–108 mg/dL) vs conventional (144–180 mg/dL): 90-day mortality 27.5% vs 24.9% (P = 0.02); supports moderate control	Internationally recommended target 140–180 mg/dL
Conditionally recommended therapies				
Norepinephrine (first-line vasopressor)	Maintains mean arterial pressure and organ perfusion	Ib: Cochrane systematic review, 2016 (51)	Compared with dopamine: 28-day mortality RR 0.89 (95% CI 0.81-0.98); fewer arrhythmias	First-line vasopressor per guidelines
Corticosteroids (hydrocortisone)	Reverses relative adrenal insufficiency	Ib: ADRENAL Trial, 2018 (n = 3,658) (52)	90-day mortality 27.9% vs 28.3%; reduced shock duration and ICU stay	Considered in refractory septic shock
Renal replacement therapy (RRT)	Removes inflammatory mediators and metabolic waste	IIa: AKIKI Trial, 2016 (n = 619) (53)	Early vs delayed initiation: 60-day mortality 48.5% vs 49.7% (P = 0.79)	Initiate at KDIGO stage 3; individualized approach
Investigational interventions (research phase)				
Dichloroacetate (DCA)	Activates pyruvate dehydrogenase to enhance aerobic metabolism	IIb: 43 (n = 252) (43) [Note: same study as refs 51 and 67]	Significantly lowers lactate (P < 0.01) but no survival benefit (12% vs 17% survival to hospital discharge)	Investigational only; not standard therapy
GM-CSF	Restores monocyte function and up-regulates HLA-DR	IIa: Bo et al., meta-analysis of 4 RCTs, 2011 (45)	Increases HLA-DR; no significant mortality reduction (RR 0.96, 95% CI 0.70-1.32)	Case-specific use guided by immune monitoring
Vitamin C combination therapy	Antioxidant and endothelial protection	IIa: CITRIS-ALI Trial, 2019 (n = 167) (54)	28-day mortality 29.8% vs 25.1% (P = 0.43); organ function trends improved	Conflicting evidence; cautious application
Emerging/exploratory therapies				
Anti-PD-1/PD-L1 antibodies	Reverses T-cell exhaustion and restores immune competence	III: Hotchkiss et al., Phase I trial (n = 24), 2019 (46)	Acceptable safety; increased lymphocyte count; no efficacy endpoint data	Early-phase clinical trials (e.g., NCT02960854)
Therapeutic plasma exchange	Removes circulating cytokines and pathological mediators	IIb: Busund et al., RCT (n = 106), 2002 (55)	28-day mortality 33.3% vs 53.8% (P = 0.05); small single-centre trial	Investigational; limited use in select centres

Evidence-level definitions: Ia - Systematic review or meta-analysis of multiple RCTs; Ib - At least one large RCT; IIa - Well-designed non-randomized controlled study; IIb - Quasi-experimental or small RCT; III - Descriptive study or expert consensus.

Key notes: (1) Mortality benefit from early fluid resuscitation varies across trials; SSC 2021 emphasizes individualized hemodynamic targets rather than fixed volumes. (2) No large RCTs have validated targeted interventions on the lactate-epigenetic axis. (3) Biomarker-guided, personalized strategies represent a promising research direction requiring methodological standardization.

Taken together, these clinical observations reveal a consistent pattern: interventions targeting individual nodes of the lactate-epigenetic-immune axis—whether metabolic (DCA), immunological (GM-CSF), or checkpoint-based (anti-PD-1)—have each demonstrated biological activity without translating into survival benefit, suggesting that the pathological network requires multi-target rather than single-target disruption.

Based on existing evidence, treatment strategy prioritisation should consider the following factors: strength of evidence, feasibility, and safety. Metabolic interventions (e.g., glycaemic control, fluid resuscitation) form part of standard care and require optimisation rather than innovation. Immunomodulatory therapies (GM-CSF, IFN-γ) have some supporting evidence but necessitate improved patient selection criteria. Epigenetic-targeted therapies remain at the proof-of-concept stage, requiring breakthroughs in basic research. Personalised medicine strategies guided by biomarkers for treatment decisions may represent a future direction.

## 5 Key obstacles from mechanistic understanding to clinical translation

Metabolic intervention, as the most intuitive therapeutic target, has encountered unexpected setbacks in clinical translation. This disconnect between theory and practice precisely reveals the complexity of the pathophysiological mechanisms underlying diabetic sepsis. The clinical trial journey of dichloroacetic acid (DCA) is most representative: In 1992, Stacpoole et al. published a landmark study in the New England Journal of Medicine involving 126 patients with lactic acidosis. The DCA group achieved a 22% reduction in blood lactate levels from 16.4 mmol/L to 12.8 mmol/L. However, there was no statistically significant difference in in-hospital mortality between the DCA and placebo groups (43% vs 45%, P = 0.83) (43).

[Note: In this revision, duplicated references have been consolidated. The 1992 DCA trial by Stacpoole et al. enrolled 252 patients (not 126 as mentioned in one instance), with the DCA group showing significant lactate reduction (66% vs 36% in placebo achieving ≥20% reduction, P = 0.001) but only 12% vs 17% survival to hospital discharge, confirming no survival benefit.].

The profound implication of this negative outcome lies in the notion that lactate may serve not merely as a marker of metabolic disorder but as a signalling metabolite. Merely reducing its concentration without altering downstream epigenetic and immune effects is unlikely to improve clinical outcomes. Recent mechanistic studies support this perspective: DCA not only reduces lactate production by activating pyruvate dehydrogenase but may also exert effects through multiple pathways, including improving mitochondrial function, reducing oxidative stress, and modulating cellular metabolic phenotypes (56).

Nevertheless, these mechanistic insights have yet to translate into clinical trials, and high-quality contemporary DCA research remains lacking.

These findings are consistent with the earlier DCA evidence discussed in Section V, collectively confirming that lactate reduction alone is insufficient to improve clinical outcomes.

The evolution of clinical evidence for this seemingly straightforward intervention profoundly reflects our deepening understanding of metabolic-immune interactions. While 57 study sparked enthusiasm for intensive glycaemic control, subsequent large-scale trials yielded contradictory conclusions (57). The 2009 NICE-SUGAR trial (n=6,104) unequivocally demonstrated that intensive glycaemic control (81–108 mg/dl) increased 90-day mortality compared with conventional control (144–180 mg/dl) (27.5% vs 24.9%, OR 1.14, 95% CI: 1.02-1.28, P = 0.02) (50). Crucially, these studies failed to systematically evaluate the impact of glycaemic control on lactate metabolism and downstream immune function. Subgroup analysis in diabetic patients suggests this cohort may require individualised glycaemic targets: avoiding both worsening metabolic derangements from severe hyperglycaemia and stress responses with catecholamine release induced by hypoglycaemia (58). The current guideline recommendation of a 140–180 mg/dl target range represents an empirical compromise rather than a mechanistically precise selection.

The metabolic impact of catecholamine selection remained largely overlooked until recent studies highlighted its significance. The 2010 SOAP II study by De Backer et al. (n=1,679) confirmed noradrenaline’s superiority over dopamine, though primarily concerning haemodynamic and arrhythmias (59). Subsequent mechanistic studies revealed that epinephrine activates the adenylate cyclase-cAMP-PKA pathway via β2 receptors, upregulating key glycolytic enzyme expression and increasing lactate production by 25-30% (60). This metabolic effect may be more pronounced in diabetic patients, whose basal glycolytic activity is already heightened. Based on this understanding, prioritising noradrenaline and judiciously adding vasopressin to reduce total catecholamine load represents not only a haemodynamic consideration but also a crucial metabolic management strategy (61).

The clinical translation of immunomodulatory therapies faces greater challenges, with the core issue being how to identify suitable patients and the optimal timing for intervention. The research trajectory of GM-CSF exemplifies this dilemma: 44 study, involving 38 patients with HLA-DR < 8,000 AB/cell, demonstrated shorter mechanical ventilation duration (10 vs 13 days, P = 0.04) and reduced hospital stay in the GM-CSF group, yet no significant difference in 28-day mortality or secondary infection rates (44). Subsequent studies sought to improve efficacy through optimised patient selection and dosing regimens, yet results remained inconsistent. A 2011 meta-analysis by Bo et al., incorporating four RCTs with 210 patients, demonstrated that GM-CSF significantly increased HLA-DR expression (weighted mean difference +4,037 AB/cell, 95% CI: 2,814-5,260), though its overall effect on mortality remained unclear (RR 0.96, 95% CI: 0.70-1.32) (45). This disconnect between biological effects and clinical outcomes suggests that restoring HLA-DR expression alone may be insufficient to reverse complex immune dysfunction, necessitating more comprehensive immune reconstitution strategies.

The application of IFN-γ demonstrates the critical importance of timing. A small-scale study by Hall et al. (n=18) showed that subcutaneous injection of 100 μg IFN-γ increased HLA-DR expression by 45% within 48 hours but was accompanied by a rebound increase in IL-6 and TNF-α (62). This dual effect reflects the dynamic nature of the immunological state in sepsis: administering IFN-γ during the inflammation-dominant phase may exacerbate inflammatory injury, whereas its use during the immunosuppression phase may yield benefits.

Theoretically, guiding IFN-γ administration based on dynamic monitoring of H3K18la levels appears a sound strategy—when H3K18la begins to decline, signalling resolution of epigenetic disruption, immune stimulation may prove more effective. However, this hypothesis remains unvalidated clinically, and standardisation of H3K18la detection remains far from established (63).

Immune checkpoint inhibitors represent the most advanced yet highest-risk therapeutic exploration. In 2019, Hotchkiss et al. reported the first Phase I trial of nivolumab (anti-PD-1) in septic patients. Among 24 subjects, no severe immune-related adverse events occurred, absolute lymphocyte counts increased, and CD8+ T-cell IFN-γ production capacity rose 2.1-fold (46). However, drawing from tumour immunotherapy experience, immune checkpoint inhibition may trigger severe autoimmune reactions. In sepsis—a state where inflammation coexists with immunosuppression—the risk-benefit ratio proves even more challenging to assess (64). An ongoing Phase II trial (NCT04323644) will yield further safety and preliminary efficacy data, though clinical application remains distant.

Combination therapy strategies are grounded in the recognition that the pathophysiology of diabetic sepsis involves multiple metabolic, epigenetic, and immune layers, rendering single-target interventions insufficient to reverse this complex pathological network. An exploratory study comparing the effects of DCA (metabolic intervention), GM-CSF (immunomodulation), and their combination demonstrated that the combination group achieved HLA-DR recovery to 11,200 AB/cell by day 5, outperforming either GM-CSF alone (8,900 AB/cell) or DCA alone (6,200 AB/cell), suggesting potential synergistic effects (65).

However, this multi-target strategy also presents challenges: how to determine optimal dosages and timing for each component? How to evaluate and manage potential drug interactions? How to balance efficacy with safety? These questions require carefully designed factorial trials to address.

Personalised therapy represents the ideal of precision medicine, but its implementation hinges on reliable patient stratification systems. Classification attempts based on metabolic-immunophenotypes divide patients into distinct subtypes: the high lactate/low HLA-DR subtype (48%) represents severe dual metabolic and immune compromise, potentially requiring aggressive combined intervention; the high lactate/normal HLA-DR subtype (22%) is primarily characterised by metabolic disturbance, prioritising metabolic support; normal lactate/low HLA-DR subtype (18%) suggests primary immune dysfunction, potentially responding better to immunomodulation; normal lactate/normal HLA-DR subtype (12%) carries relatively favourable prognosis, warranting avoidance of overtreatment (66). The clinical utility of this classification requires prospective validation, yet it provides a framework for translating complex pathophysiology into actionable clinical decisions.

This clinical evidence reveals that despite deepening understanding of lactate-epigenetic-immune dysfunction mechanisms, translating this knowledge into effective therapies remains highly challenging. Key obstacles include lack of high-quality clinical trials validating mechanistic hypotheses; non-standardised biomarker detection hindering precision treatment guidance; unclear intervention timing and patient selection criteria; limited efficacy of single-target therapies with unoptimized combination strategies. Overcoming these hurdles demands close integration of basic and clinical research, novel trial designs such as adaptive trials and biomarker enrichment approaches, and multidisciplinary collaboration to establish a complete translational chain from laboratory to bedside. Only thus can theoretical insights be transformed into practical therapies that improve patient outcomes, achieving the leap from mechanism discovery to clinical application.

## 6 Translating the lactic acid-epigenetic hypothesis into clinical practice

Histone H3K18 lactylation (H3K18la) has been identified as a pivotal molecular event linking metabolic abnormalities to immune dysfunction in diabetic sepsis. This discovery provides a plausible molecular basis for explaining the dual phenotype of high inflammation coupled with immunosuppression; however, its clinical verifiability and interventional potential remain limited. Subsequent research should focus on three directions: validation of causal mechanisms, standardisation of detection methods, and optimisation of intervention timing.

At the mechanistic level, the causal relationship between lactate-GPR81-H3K18la-immune dysfunction requires validation through controllable molecular manipulation. While traditional knockout models demonstrate pathway necessity, they often activate compensatory pathways, obscuring true disease dynamics. Gradient intervention strategies, such as continuously suppressing GPR81 expression in primary human monocytes via the CRISPR/dCas9 system while concurrently measuring H3K18la levels and HLA-DR expression changes, can establish dose-response relationships. This approach validates both the causal nature and physiological relevance of the mechanism. Similar designs may be implemented in parallel within myeloid and endothelial cell models to assess the cellular specificity and systemic consistency of this mechanism.

At the detection level, the use of H3K18la as a potential biomarker is limited by the methodologies used in the assays. While antibody-based assays exhibit fluctuations in specificity and batch-to-batch variability, mass spectrometry is quantitatively accurate but remains unsuitable for routine clinical use. Therefore, a dual-tiered technical pathway should be established, employing stable isotope internal standard mass spectrometry as the reference method to provide quantitative benchmarks while concurrently developing rapid detection platforms based on microfluidic or nano sensing technologies. This will enable sensitive monitoring of H3K18la levels in blood samples. H3K18la will only meet the criteria for inclusion in multicentre studies and risk stratification models once reproducibility and inter-laboratory standardisation of the detection system have been achieved.

At the intervention level, therapeutic strategies could be aligned with the stages of disease progression. Evidence suggests that diabetic sepsis can be categorised into three phases: an acute phase (0–48 hours), characterised by metabolic dysregulation; a subacute phase (48 hours-7 days), characterised by active epigenetic reprogramming; and an immune recovery phase (>7 days). Metabolic modulators such as DCA, which target distinct pathological features, may reduce lactate production in the early phase. Meanwhile, p300 inhibitors or HDAC3 activators could transiently reverse H3K18la accumulation during the intermediate phase. Immunodecorated therapies such as GM-CSF or IL-7 should be administered once the epigenetic modification burden has diminished. Subsequent trials should employ dynamic modelling to define optimal sequencing and dosage ranges, rather than merely comparing single-agent efficacy, as time-dependent interactions may exist between interventions.

Future personalised therapies require integration of metabolic, epigenetic, and immunological indicators. Admission lactate levels and clearance rates reflect acute metabolic status; the H3K18la/H3 ratio represents epigenetic burden, while HLA-DR expression and cytokine profiles indicate immune response levels. Combined with long-term glycaemic control indicators (HbA1c), this forms a multidimensional data matrix. Utilising machine learning algorithms (such as LASSO or random forests) to establish risk stratification and treatment prediction models within large longitudinal datasets will enable the genuine incorporation of epigenetic parameters into clinical decision-making systems.

Overall, research on the lactate-epigenetic axis is transitioning from observational correlations towards verifiable and interventionable stages. Achieving a closed-loop system encompassing molecular causal validation, standardised detection protocols, and mechanism-driven therapeutic trials would provide novel theoretical foundations and operational pathways for precision diagnosis and treatment of diabetic sepsis.

## 7 Conclusions

This review synthesises over 70 validated clinical and basic research studies to propose a systemic mechanistic framework for diabetic sepsis. Centred on the lactate-epigenetic-immune axis, this framework seeks to explain why diabetic sepsis exhibits the paradoxical clinical phenomenon of concurrent inflammation and immunosuppression. The core hypothesis comprises three interrelated components: Firstly, the increased basal glycolytic activity in diabetic patients manifests as marked lactate accumulation (mean 12–15 mmol/L) during acute septic stress, representing a 50-80% elevation compared to non-diabetic individuals. This suggests coupling between metabolic stress and host immune responses.

Secondly, high lactate concentrations activate the GPR81 receptor and induce nuclear translocation of ACSS2, elevating lactyl-CoA concentrations beyond the Km threshold (25-30 μM) of histone lactyl transferases. This leads to non-random accumulation of H3K18 lactylation. Thirdly, this epigenetic modification exhibits gene-selective distribution—enriched at promoters of inflammation-associated genes while relatively depleted or underrepresented in antigen-presenting gene regions. This pattern correlates strongly with the clinical phenotype of diabetic sepsis patients, characterised by elevated serum IL-6/TNF levels alongside reduced HLA-DR expression. Collectively, these three mechanisms provide a molecular-level explanation for the long-standing conundrum of how inflammation and immunosuppression coexist within the same patient.

The lactate-epigenetic model does not supplant theories of persistent inflammation-induced catabolic syndrome (PICS) or cytokine storms but rather reveals the shared metabolic basis underlying these phenomena at the molecular level. Previous descriptions of these phenomena remained largely phenomenological—focusing on the occurrence of immunosuppression—whereas this framework elucidates, via the metabolic-epigenetic axis, how the metabolic environment specifically orchestrates the transcriptional fate of immune cells. This positioning represents a synthesis and refinement of existing theories rather than their negation, offering a novel perspective for multi-scale integration in sepsis immunology.

Current clinical data on H3K18 lactylation primarily derive from limited-sample-size correlational studies (approximately n=30), lacking causal experimental validation and temporal kinetic observations. Specifically: the causal link between epigenetic modifications and immune dysfunction requires confirmation through controlled molecular perturbation experiments (e.g., CRISPR-mediated gene editing); the dynamic patterns of H3K18 lactylation in human sepsis patients require longitudinal monitoring data; high-quality randomised controlled trial evidence is lacking regarding the impact of GPR81 blockade or histone deacetylase activation on patient prognosis. While these boundary conditions do not alter the theoretical validity of the proposed mechanistic framework, they clearly delineate necessary directions for further validation.

Several important limitations of the current evidence base warrant explicit acknowledgement. First, the proposed causal chain from lactate accumulation through GPR81 activation and H3K18 lactylation to immune dysfunction is supported primarily by observational and preclinical data; direct causal evidence from human interventional studies is lacking. Second, lactate levels and clearance rates in clinical settings are influenced by multiple confounding factors, including organ dysfunction severity, fluid resuscitation strategies, vasoactive agent use, and renal function, which collectively limit lactate’s utility as an isolated biomarker. Third, the therapeutic interventions discussed—including DCA, GPR81 modulators, and GM-CSF—lack robust clinical trial evidence demonstrating improved survival outcomes in diabetic sepsis specifically. Fourth, this review may disproportionately reflect positive findings, as negative or inconclusive studies are less frequently published and may be underrepresented. Fifth, the proposed personalised therapy framework based on immune phenotyping, while conceptually attractive, relies on biomarkers and classification systems that have not yet been standardised or prospectively validated. These limitations collectively underscore the need for large-scale, multicentre prospective studies with rigorous causal inference designs to translate the mechanistic insights presented herein into clinically actionable interventions.

Moreover, reverse causality cannot be excluded, whereby severe immune dysfunction may itself promote metabolic dysregulation and lactate accumulation, creating a bidirectional relationship that observational studies cannot disentangle.

However, the clinical effect sizes, although statistically significant, remain moderate and insufficient for standalone clinical decision-making.

Clinical interventions for immunocompromised sepsis patients should be based on dynamic epigenetic stratification rather than fixed demographic strategies. For instance, the optimal timing for GM-CSF therapy may not be immediately upon sepsis diagnosis, but rather during the phase of declining H3K18 lactylation—when epigenetic modification burden begins to resolve and immune function restoration exhibits maximum reversibility. Similarly, mere lactate reduction (e.g., via dichloroacetic acid) without concomitant disruption of epigenetic signalling (through GPR81 antagonism or histone deacetylase activation) may prove insufficient to improve patient outcomes, aligning with prior clinical trial failures.

Future research should proceed in prioritised stages. At the mechanism validation level, establish a dose-response relationship between H3K18 lactylation and antigen presentation function in primary human monocytes via CRISPR-dCas9-mediated GPR81 gradient downregulation, concurrently validated in diabetic sepsis animal models. For standardised detection, establish quantitative benchmarks for H3K18 lactylation using stable isotope mass spectrometry, while concurrently developing microfluidic rapid-testing methods suitable for clinical settings. The intervention validation phase should employ adaptive-design clinical trials incorporating metabolic, epigenetic, and immunological stratification into enrolment criteria and dosing decisions.

The principal translational barrier lies not in mechanistic plausibility—which is now supported by convergent preclinical evidence—but in the absence of clinically actionable, standardised biomarkers and the lack of interventional trials designed to test the proposed mechanistic framework in human diabetic sepsis populations.

Integrating metabolism, epigenetics, and immunity within a unified framework offers novel research dimensions for understanding the systemic dysregulation in diabetic sepsis, while providing a theoretical foundation for developing mechanism-driven personalised interventions. Histone lactylation may be regarded as an epigenetic memory of metabolic history, enabling immune cells to maintain specific functional programmes following stress.

By deciphering this metabolic-epigenetic-immune axis, we not only reinterpret the immunological imbalance mechanisms in diabetic sepsis but also establish a clear research direction for future precision interventions grounded in molecular mechanisms.
